# A Land Space Development Zoning Method Based on Resource–Environmental Carrying Capacity: A Case Study of Henan, China

**DOI:** 10.3390/ijerph17030900

**Published:** 2020-02-01

**Authors:** Xiaotong Xie, Xiaoshun Li, Weikang He

**Affiliations:** 1 Jiangsu Key Laboratory of Resources and Environmental Information Engineering, China University of Mining and Technology, Xuzhou 221116, China; xxtong0120@163.com (X.X.); hewk@cumt.edu.cn (W.H.); 2 Research Center for Transition Development and Rural Revitalization of Resource-based Cities in China, China University of Mining and Technology, Xuzhou 221116, China; 3 Observation and Research Station of Jiangsu Jiawang Resource Exhausted Mining Area Land Restoration and Ecological Succession, Ministry of Education, Xuzhou 221116, China

**Keywords:** land space, resource–environmental carrying capacity (RECC), three-dimensional magic cube evaluation model, development zoning, Henan, China

## Abstract

As a key element in China’s spatial planning, the development zoning of land space has become a focus of China’s current activity. During its rapid social and economic development, China has faced severe and diverse challenges regarding sustainable development, such as farmland occupation, environmental degradation, urban land disorder expansion, etc. Against this backdrop, research on the linkage between resource–environmental carrying capacity (RECC) and the development zoning of land space in the process of sustainable development has received increased attention, and an accurate evaluation of the RECC would provide useful guidance for Chinese policy makers to carry out the development zoning of land space. This paper uses Henan Province as an example to construct a comprehensive evaluation model of “resource carrying capacity (RCC)–eco–environmental carrying capacity (EECC)–socio–economic carrying capacity (SECC)”, which calculates the level of RECC in a provincial area. In addition, this paper designs a correlation model between the RECC and the development zoning of land space, which uses a three-dimensional magic cube evaluation model to analyze the development zoning layout of land space. The results showed that a geographical pattern exists, where in the southwestern areas of Henan Province have a higher RECC than the central and northeastern areas. The results also indicated that the land space patterns of Henan Province can be divided into seven types of areas through a three-dimensional magic cube evaluation model, which can better reflect the spatial differentiation characteristics of the comprehensive index of RECC. The results of this study offer an important reference for policy-makers to make decisions and also provide a scientific and pragmatic basis for the formulation of sustainable development strategies.

## 1. Introduction

Industrialization and urbanization have been an important feature in the process of human development throughout history [1]. Due to economic development and population growth, China’s land space per capita decreased by two and a half times, and the amount of land under cultivation per capita was also cut in half, over a 65-year period [2]. With the rapid development of China’s industrialization and urbanization, all land is becoming scarce due to competing demands for its use, and sustainable land use has come under increasing pressure from industrialization, urbanization and ecological civilization construction [2]. Therefore, whether the development of land space can be matched and coordinated with the limited resource environment without damaging the background of the resource environment has become a focal point in many social and academic circles. In light of the problems of the imbalanced land space development and tight resources in many areas of China, the “National Land Planning Outline (2016–2035)”, issued by the State Council in 2017, sets the “survival line” for land development. Meanwhile, the establishment of a spatial planning system has become a major national strategy, emphasizing that the evaluation of resource–environmental carrying capacity (RECC) should be the premise and basis of spatial planning. Therefore, handling the relationship between land space development and RECC and valuing land space development goals under the constraints of resource and environmental carrying capacity are significant factors for China’s sustainable development.

The rational development of land space is a key issue in the process of a nation’s sustainable development, and research on the RECC forms the basis of land space development. Carrying capacity is an important concept used to measure the relationship between human economic and social activities and the natural environment [3]. The idea of carrying capacity was first applied in biology, population biology, and ecology, and its application range was gradually extended to the environmental field. In this way, the concept of carrying capacity was given more precise meanings [4], such as land resource carrying capacity [5,6], water resource carrying capacity [7,8], resource carrying capacity [9,10], ecological carrying capacity [11,12], urban carrying capacity [1,13], RECC [14,15], and so on. The concepts of RECC were initially derived at different times and were largely employed within the resource and environmental fields. However, the research history of land resource carrying capacity and water resource carrying capacity is long and numerous results have been obtained [15,16]. Research on RECC has mainly focused on the regional carrying capacity level [17], carrying capacity monitoring and warning [18], and single factor carrying capacity evaluation [19]. The future research trends of RECC will likely shift from single factor resource environment analysis to a comprehensive factor analysis of resources and the environment [20]. Moreover, the RECC will become the basis for primary functional area planning and spatial planning in the future [21].

From the perspective of the RECC method, one of the basic elements of the RECC is to build an index system. The index system of carrying capacity is established first, followed by determining the weight ascribed to the indicators, and finally completing the evaluation by various evaluation models [22,23]. The construction of an index system usually requires the resources, environment, ecology, society, economy, and different systems to be restricted and influence each other. In the construction of an index system, researchers usually rely on three methods. The first method constructs an index system that includes a single factor, such as resources, the environment, the economy, and other criteria [24]. The second constructs an index system of pressure-state-response [25]. The third constructs support-pressure as the system layer [15]. Several models, such as the system dynamics model [26,27], ecological footprint method [11,28], GIS (Geographic Information System) analysis method [29], energy analysis method [30], and comprehensive evaluation method [20], have been applied to the evaluation of RECC.

In developed countries, land space control is one of the major governmental regulatory measures for orderly regional development [31]. In order to control the development of land space, the Chinese government has focused on the planning and development zoning of land space in recent years [32]. At present, research on the development zoning of land space in China has become an important topic. For example, Jiang [2] proposed a new zoning method for classifying and dividing agricultural water and land resources and studied a larger area, the Northwest Arid Region of China, which provides a guide for the zoning of water and land resources. Fan [33] used a new land use function classification system based on multi-source data and applied the system to assess 12 land use functions in the Jiangsu Province of eastern China. The development zoning of land space is a scientific concept used to handle space control and space development and is an important tool for spatial planning and sustainable development. The development zoning of land space is mainly dependent on the concept of land space and is similar to the concept of land space utilization. Some issues related to the development zoning of land space, such as the intensity of land space development [34,35], land space development allocation [36,37], and spatial planning [38], were discussed. Moreover, from the viewpoint of the linkages between RECC and land space planning, Yue and Wang [39] explored the logical relationship between RECC and land space planning and discussed the model of RECC for land space planning. Wu [40] proposed land space optimization and utilization strategies from the perspective of ecological–production–living space. Zhou [41] designed a framework using system science, the entropy weight method, the triangle model, and the coupling coordination degree model for land use multi-functionalization assessment.

In summary, the current research mainly aims at single factor research on carrying capacity or the use of different models and methods to discuss the development layout of land space [42,43,44], seldom linking the study of RECC with land space planning or zoning. Meanwhile, research on land space development zoning based on the spatial differentiation of regional RECC is still rare, and there is no sufficient empirical research at the provincial level. With the continuing emphasis on spatial planning in China, the RECC will become an important link in planning evaluation. In addition, analyzing the regional differences of land space based on the RECC is crucial to addressing China’s national and regional development and sustainability goals.

Based on this, we developed a three-dimensional magic cube evaluation model that would enable us to extend existing studies on the relationship between RECC and land space zoning. The three-dimensional magic cube evaluation model in this study makes it possible to analyze provincial land space zoning in the sustainable development process. This research intends to fulfill three objectives. First, this study aims to analyze the spatial patterns of RECC at the provincial level, using a new comprehensive index system from the perspectives of resource carrying capacity (RCC), eco–environmental carrying capacity (EECC), and socio–economic carrying capacity (SECC). Second, this paper aims to construct a suitable land space zoning model to assess land space multi-functionalization using a three-dimensional magic cube evaluation model to measure the relationship between the RECCs of different evaluation units and the development zoning of land space. Thirdly, this study explores the mode and principle of land space function zoning suitable for sustainable development and presents some zoning suggestions for land space development at the provincial level.

## 2. Theoretical Analysis of RECC and Land Space

RECC, as the link between the social system, environmental system, and economic system, is the key to coordinating the population, resources, and the environment; RECC is also an important foundation for sustainable development. There are many definitions of RECC. However, researchers generally agree that RECC is an important criterion for evaluating the coordination degree of resource environments and social economics. Achieving sustainable development is the ultimate goal that planners, managers, and policy makers seek. Thus, addressing the needs of resources and the environment poses a great challenge for RECC research [1]. The evaluation of RECC has changed from single resource evaluation to comprehensive evaluation, and the evaluation methods have been continuously enriched. Meanwhile, the evaluation objects have focused mostly on typical regions [37]. In practice, the evaluation of RECC in most regions remains at the strategic guidance level, and the support for optimizing land space zoning is insufficient [21].

**Figure 1:**
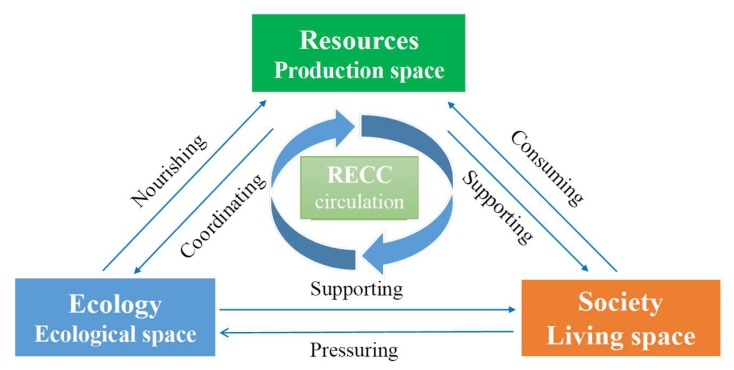
Research framework of the resource–environmental carrying capacity (RECC) and land space.

The concept of land space zoning generally refers to the comprehensive division of an area within a certain range. Europe is the birthplace of land space zoning, and its ideas of land space function zoning can be traced back to the end of the 18th century and the beginning of the 19th century [45]. Land space function is the basis for describing the status of land use in a certain area. Thus, land space generally provides the follows three functions: a production function, living function, and ecological function. These three functions are interrelated [46]. In other words, the function of land space is generally divided into the three aspects of agricultural production (production), urban development (living), and ecological protection (ecology) to ensure the rational development of space resources (Figure 1). The RECC can provide valuable evaluation methods and measurable indicators for assessing the land space function of a region. If the population and economy gradually increase and exceed the threshold of the carrying capacity, there will be negative impacts on the function of land space [47]. As an important basis for guiding spatial resource allocation for sustainable development, the assessment of RECC can support the application needs of land space development. Therefore, in China, a comprehensive evaluation of the RECC for different regions is carried out. Meanwhile, according to the different types of areas of RECC, it is possible to reasonably realize the development zoning of land space and ensure the optimal allocation of land resources.

The RECC system can be divided into three aspects—resources, ecology, and society—which have a circular relationship. Similarly, land space can also be divided into the three aspects of production space, ecological space, and living space. The two systems of RECC and land space thus correspond to each other (Figure 1). Therefore, establishing the corresponding relationship between the RECC and the development zoning of land space could provide a beneficial reference for the rational division of land space.

## 3. Materials and Methods

### 3.1. Study Area

**Figure 2:**
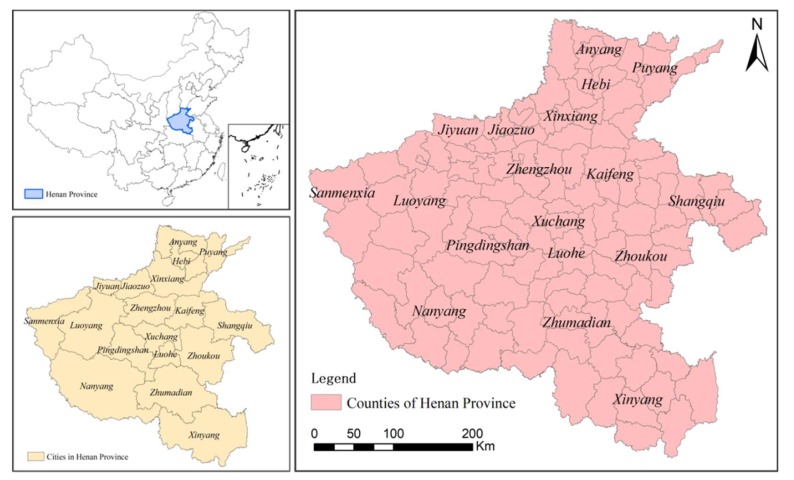
The study area.

Henan Province is situated in the middle of China and the middle and lower reaches of the Yellow River (Figure 2). It has an area of 167,000 km^2^ and a total population of 109.06 million, with rich resources and comparatively high levels of socio–economic development. In 2016, the GDP (Gross Domestic Product) of the province was 4.02 trillion Yuan, and the growth rate of major economic indicators was higher than the national level. Meanwhile, the urbanization level was 48.5%. Henan Province is composed of plains and basins, mountains, and hills, accounting for 55.7%, 26.6%, and 17.7% of the total area, respectively. Henan Province belongs to the middle ground of China’s economic development from east to west and is an important hub for the comprehensive national transportation network. Meanwhile, Henan Province governs 17 municipalities directly under the Central Government, one city under direct provincial administration, 20 county-level cities, and 84 counties. By placing emphasis on the strategy of “the rise of the central plains”, the economy of Henan Province has achieved steady and rapid development. However, at the same time, the area’s resources and environment have been damaged to some extent. Based on this phenomenon, Henan Province has gradually realized a transition from the pure pursuit of economic development to the coordinated development of resources, ecology, and economy. As one of the nine provincial-level spatial planning pilots established by the central government, Henan Province has been used for research on the development zoning of land space under the constraints of RECC, which has important guiding significance for the improvement of spatial planning and sustainable development. Above all, with limited land resources, the issues of the rational division of land space and of coordinating the conflict between production space (resources), ecological space (ecology), and living space (society and economy) have become major tasks which are necessary to achieve the sustainable development of Henan Province in the future. Therefore, this study provides a beneficial and timely land space development reference for land planners and policymakers.

### 3.2. Data Sources

This study takes the counties of Henan Province as the research unit and uses data from 2016. The water resource data were obtained from the water resources bulletins of Henan Province and cities in 2016 [48]. The environmental data were obtained from the environmental status bulletin of Henan Province and cities in 2016 [49]. The other data were obtained from the Henan Statistical Yearbook 2017 and the other cities’ Statistical Yearbooks for 2017 [50], as well as the statistical bulletin on national economic and social development of Henan and cities in 2016 [51].

### 3.3. Research Methods

#### 3.3.1. Construction of an RECC Evaluation Index System

In the research of an RECC, we must fully consider the feedback and interaction between the bearer object and the hosted object when selecting indicators. RECC should consider how resources, the environment, and human activities interact. Therefore, on the basis of the above principles, we reference the sustainable development index system of the Chinese Academy of Sciences combined with the actual situation of Henan Province. Meanwhile, from the perspective of RCC, EECC, and SECC, 22 indicators were determined. The results are shown in Table 1.

The RCC index mainly reflects resource abundance. This index includes the components of resources, such as construction land, cultivated land, forest resources, water resources, and grain output, which represent the level and carrying capacity of the resources. The EECC index mainly reflects the abundance of eco–environmental factors and the utilization and consumption of human activities on the environment. It covers the ecological land coverage rate, green land coverage rate, atmospheric environment capacity, water environment capacity, COD (chemical oxygen demand) emissions, industrial SO_2_ emissions, and dust emissions, which represent the overall quality of the ecological environment and the pressure of human activities on the ecological environment. The SECC index mainly reflects the coordinated development of the society, economy, and population, as well as the consumption of resources by social and economic activities.

#### 3.3.2. Dimensionless Standardization

Considering the differences that exist in both the dimension and magnitude of each of the selected indicators, the data need to be normalized before an analysis can be undertaken. The indicators are grouped into two types: “positive indicators” and “negative indicators” (Table 1). The positive indicators refer to the indicators for improving RECC with an increasing value. In contrast, the negative indicators refer to a deteriorating RECC with an increasing value [1], such as industrial SO_2_ emissions, dust emissions, etc.

For the “positive indicators”, the data are transformed by Equation (1):

(1) $$r_{ij}=\frac{x_{ij}-\min(x_{ij})}{\max(x_{ij})-\min(x_{ij})}$$

For the “negative indicators”, the data are transformed by Equation (2):

(2) $$r_{ij}=\frac{\max(x_{ij})-x_{ij}}{\max(x_{ij})-\min(x_{ij})}$$

where *r_ij_* is the standardized value of the *j* index in the *i* region (*i* = 1, 2, …, *m, j* = 1, 2, …, *n*), *m* is the number of regions evaluated, and *n* is the number of indicators evaluated. Meanwhile, max (*x_ij_*) and min (*x_ij_*) represent the maximum and minimum value of indicator *j* in the *i* region. All index values are within the scope of (0,1) after treatment.

#### 3.3.3. Determination of Indicator Weight

Considering the complexity of the resource–environment–society system and the uncertainty of the RECC index, in order to increase the objectivity of the carrying capacity evaluation, the entropy method was used to determine the index weight [52,53]. The entropy method is an objective weighting method. It determines the weight of the index by calculating the information entropy, and an index with a large degree of variation has a large weight. Meanwhile, this method is widely used in various fields and has strong research value. Therefore, this study attempts to use the entropy method to determine the index weights according to the sample data’s degree of variation and evaluates the RECC at the county level in Henan Province. The specific steps of this method are as follows:

(1) Calculate the proportion of the indicator *j* in the region *i*.

(3) $$P_{ij}=Z_{ij}/\sum_{i=1}^{n}Z_{ij}$$

where *m* represents the number of indicators, and *n* represents the number of samples (*i* = 1, 2, …, *n*; *j* = 1, 2, …, *m*).

(2) Calculate the information entropy of indicator *j*.

(4) $$e_{j}=-k\sum_{i=1}^{n}p_{ij}\ln(p_{ij})$$

where *k* = 1/ln(n), 0 ≤ e_j_ ≤ 1.

(3) Calculate the difference coefficient of indicator *j*.

(5) $$h_{j}=1-e_{j}$$

(4) Calculate the weight of indicator *j*.

(6) $$w_{j}=h_{j}/\sum_{j=1}^{m}h_{j}$$

(5) Calculate the comprehensive evaluation value of RECC in region *i*.

(7) $$Y_{i}=\sum_{j=1}^{m}w_{ij}p_{ij}$$

According to the RECC evaluation results calculated by the entropy weight method, the evaluation results are divided into five levels using the natural break method.

### 3.4. Three-Dimensional Magic Cube Evaluation Model

In this study, a three-dimensional magic cube model [12,54] is used to associate the RECC with a land space function. The basic idea of the three-dimensional magic cube model is that the element vectors form spatial units with different functions in a three-dimensional space [54]. Each element has an exact position in three-dimensional space and has strong visibility and intuitiveness. This study applies this concept to the three-dimensional magic cube method to construct a model of the relationship between RECC and land space development zoning. By constructing a three-dimensional space, the various element indicators are classified, and the dimensional nodes and classification level are set according to the number of classifications. Then, the node attribute values are clarified, and the main functions embodied by each element are combined and classified; finally, a three-dimensional magic cube is formed.

According to the concept of a three-dimensional magic cube, this study will construct a three-dimensional space and divide each element level by means of its position, node, and dimension, thus forming different functional areas.

#### 3.4.1. Constructing a Three-Dimensional Space for RECC

**Figure 3:**
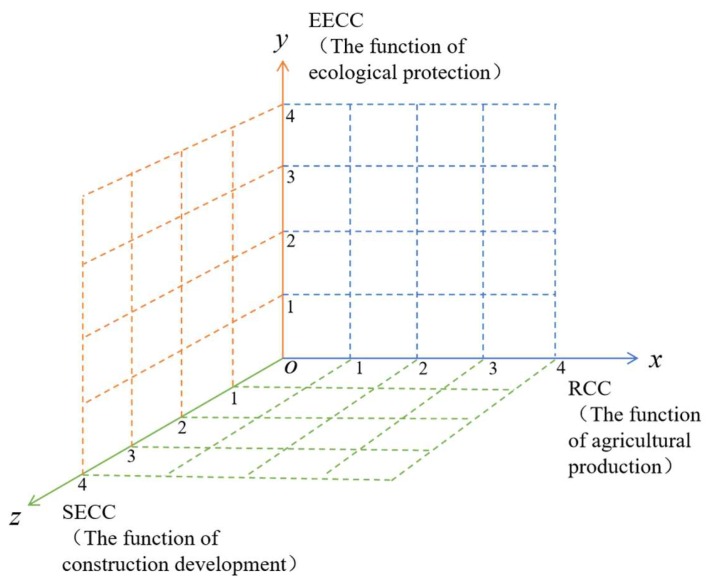
Three-dimensional magic cube evaluation model.

Based on the relationship between RECC and the function of land space, which correlates RCC, EECC, and SECC to the function of agricultural production, ecological protection, and construction development, we form a three-dimensional magic cube evaluation model corresponding to the RECC and development zoning of land space, as shown in Figure 3.

The model takes the RCC, EECC, and SECC as the x-axis, y-axis, and z-axis, respectively, of the three-dimensional space. First, nodes 1,2,3, and 4 are constructed according to the distance from the node to the origin of the three-dimensional space. At the same time, according to the relationship between the mean value and standard deviation of RECC, the four level intervals are determined. The division criteria are shown in Table 2. The larger the value of RECC, the farther its distance from the origin, and the stronger the RECC level. On the contrary, the smaller the value of RECC, the closer its distance from the origin, and the smaller the RECC level.

#### 3.4.2. Three-Dimensional Magic Cube Space Construction Principle

**Figure 4:**
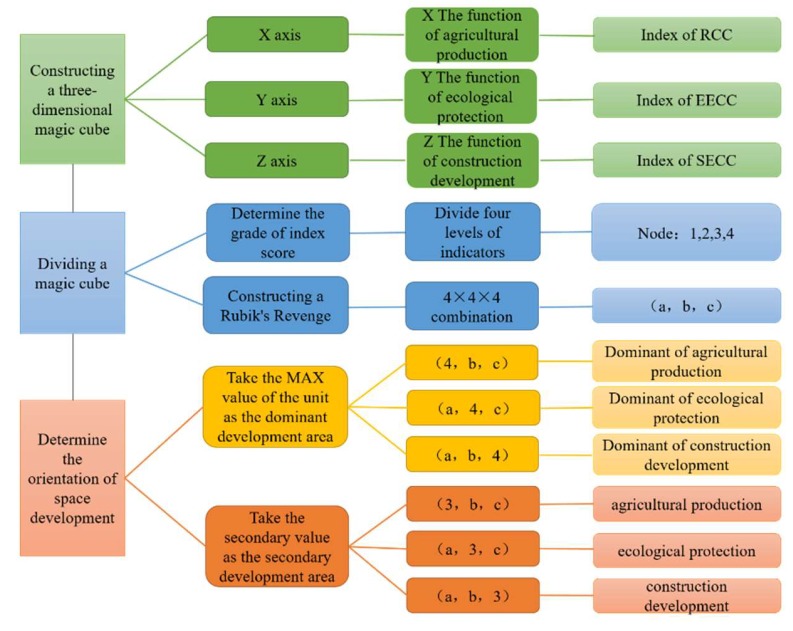
Three-dimensional magic cube space construction principle.

According to the classification level in Table 2, the three-dimensional space is divided into a three-dimensional magic cube of 4 × 4 × 4, and 64 kinds of land space function types can be obtained. In addition, the regions represented by each combination type have different functions of land space, and the magic cube combination consists of coordinates (a, b, c). At the same time, a, b, and c represent the level of agricultural production function, ecological protection function, and construction development function, respectively. The three-dimensional magic cube space construction principle is shown in Figure 4.

#### 3.4.3. Land Space Zoning Criteria

We determine the dominant development area and development area by means of the coordinate in the cube unit and the size of the unit node (Table 3). When there is only one functional category in the three-dimensional space unit coordinates (a, b, c) with a level of 4, the unit is determined to be the dominant agricultural area (A1), key ecological protective area (E1), or construction development dominant area (C1). When there is only one functional category in the three-dimensional space unit coordinates (a, b, c) with a level of 3, and the other category levels are less than 3, then the unit is determined to be the functional agricultural area (A2), functional ecological area (E1), or construction development area (C1). When the level of the functional category in the three-dimensional space unit coordinates (a, b, c) is in the interval of [1,2], the unit is determined as the potential resource area (R1). If there are two or more functional categories in coordinates (a, b, c) with the same level, the function of land space is determined according to the value of RECC and the actual situation of the region.

## 4. Results

### 4.1. RECC Evaluation

**Figure 5:**
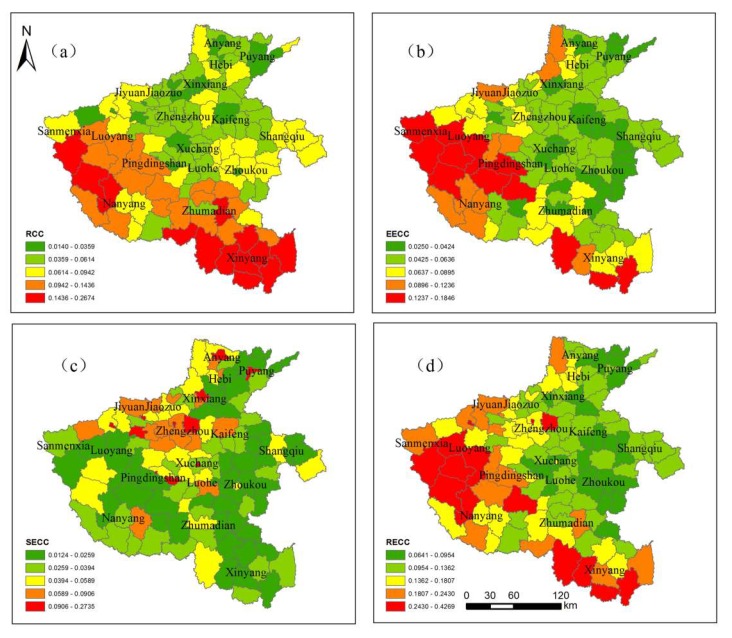
Evaluation results of (**a**) the RCC, (**b**) the EECC, (**c**) the SECC and (**d**) the RECC in Henan Province.

According to Equations (1)–(7), we calculated the single factor evaluation and comprehensive evaluation results of RECC in Henan Province and divided the results into five grades (low, lower, medium, higher, and high) by using the natural break method. The larger the value, the stronger the carrying capacity, and the smaller the value, the smaller the carrying capacity. In Figure 5a–d represent the RCC, EECC, SECC, and RECC of Henan Province, respectively. The level and spatial distribution of each type of carrying capacity are as follows.

Figure 5a reflects the spatial difference in the RCC of each county in the province. The RCC index of Henan Province is between 0.0140 and 0.2674, and the high-value areas (including the high-value and higher-value areas) are mainly distributed in most western and southern counties, and the level is between 0.0942 and 0.2674. In the western high-value area, there are many hills and a smaller population, so the resource carrying pressure is small. On the other hand, southern Henan is one of the province’s major grain producing areas, so it has a large area, abundant resources, and a relatively high carrying capacity. The low-value areas (including the low-value and lower-value areas) are mainly distributed in the most central and northern counties of Henan, and the level is between 0.0140 and 0.0614. In the low-value area, some of the areas belong to the Central Henan urban agglomeration, and the urban space and population density are large, thereby affecting the RCC. Medium value areas are mainly distributed in some eastern counties and a few southwest counties in Henan, with a level of 0.0614–0.0942.

Figure 5b reflects the spatial difference in the EECC of each county in the province. The EECC in Henan shows a gradual increase from northeast to southwest, indicating an obvious regional difference. The EECC index of Henan province is between 0.0250–0.1846, and the maximum, minimum, and mean values of the EECC are0.1846, 0.0250, and 0.0633, respectively. The spatial distribution presents a small scattered and large centralized coexistence. Specifically, high-value areas are concentrated in the counties of southwest Henan, and the level is between 0.0895–0.1846, which indicates that the western and southern regions play important roles in maintaining ecological safety. Meanwhile, low-value areas are concentrated in the central economic development area and the northern plain area of Henan Province, and the level is between 0.0250–0.0636. In this area, the urban space and population density are large, which is not conducive to the improvement of ecological environment quality. Medium-value areas are scattered in some southern and northwestern counties of Henan, and the level is between 0.0636–0.0895.

Figure 5c reflects the spatial difference in the SECC of each county in the province. The SECC index of Henan is between 0.0124 and 0.2735, of which the low-value area is obviously greater than the high-value area, which indicates that the SECC of Henan is generally in a low state. Meanwhile, the maximum, minimum, and mean values of the SECC are0.2735, 0.0124, and 0.0435, respectively. This implies that the unbalanced development of Henan is still prominent. Specifically, some central and northern counties are in the high carrying capacity area, while most of the remaining counties are in the low carrying capacity area, which suggests an obvious imbalance and greater polarization in economic development.

Figure 5d reflects the spatial difference in the RECC of each county in the province. The levels of RECC range between 0.0641 and 0.4269 and mainly present a spatial pattern of east–west differentiation. The maximum, minimum, and mean values of the RECC in Henan Province are0.4269, 0.0641, and 0.1472, respectively. Overall, the county with a high carrying capacity mainly forms a semi-circular distribution along the southwestern part of Henan, and its natural resources in this region are better. Moreover, its social and economic development causes less damage to the ecological environment, and the carrying pressure is relatively low. The county areas with a low carrying capacity are mainly concentrated in the north, central, and eastern areas of Henan; the regional population and resource development density, as well as the carrying pressure, are relatively high. Medium-value areas are scattered in some counties in northwest and southwest Henan.

### 4.2. Development Zoning Analysis of Land Space Based on a Three-Dimensional Magic Cube Evaluation Model

**Figure 6:**
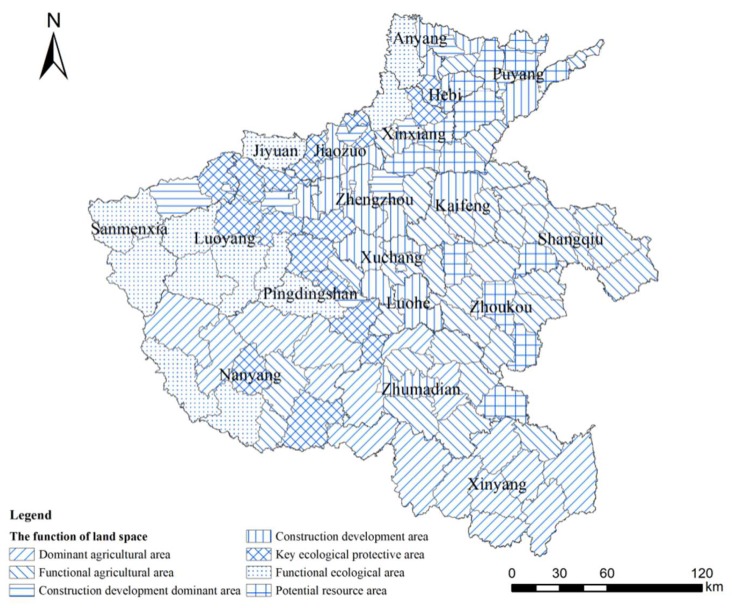
Evaluation results of land space development zoning in Henan province.

According to the division criteria of RECC in Table 2 and the land space development zoning principle in Table 3, and combined with the corresponding relationship between the results of RECC and the development zoning of land space, a functional type of land space for the evaluation unit can be obtained. Based on the current thoughts of “production–living–ecological space”, this paper divides the type of land space development into an agricultural development area, an ecological development area, a construction development area, and a potential resource area. Meanwhile, according to the actual situation of Henan Province, the type of land space development is refined, and the results have formed the land space development layout in Henan Province, as shown in Figure 6. Specifically, the agricultural development area is refined into a dominant agricultural area and an agricultural functional area; the division criteria are determined by the value of the RCC. The ecological development area is refined into the key ecological protective area and ecological functional area, and the division criteria are determined by the value of the EECC. The construction development area is refined into the construction development dominant area and construction development area, and the division criteria are determined by the value of the SECC (Figure 6).

The dominant agricultural area is mainly concentrated in the Nanyang basin and the southeastern plains. The functional agricultural area is distributed around the eastern plains and includes the counties of Xinyang city, some counties of Luohe city, some counties of Kaifeng city, and some counties of Xinxiang city (Figure 6).

The key ecological protective area is mainly concentrated in the northwest and to the south of Henan. The ecological functional area is distributed around the counties of Luoyang city, the counties of Pingdingshan city, and the counties of Nanyang city (Figure 6).

The construction development dominant area includes the cities of Zhengzhou, Luoyang, Xinxiang, Jiaozuo, Pingdingshan, and Puyang. The construction development area mainly includes the economic development zone of central, northern, and eastern Henan (Figure 6).

The characteristics of the potential resource area are mainly manifested in its low development density and ecological optimization degree, but its development direction is unclear. Meanwhile, when resources in some key development areas are depleted, the potential resource area may have certain advantages. The potential resource area primarily includes some counties in Anyang city, some counties in Xinxiang city, some counties in Puyang city, and some counties in Zhoukou city (Figure 6).

### 4.3. Land Space Development Zoning Characteristics and Development Suggestions

The development zoning of land space entails a comprehensive study of resources from the three aspects of RCC, EECC, and SECC. Different development zoning areas have obvious influencing factors and development characteristics. The following will be based on the development zoning types of land space alongside some reasonable suggestions for the development layout of land space.

**Figure 7:**
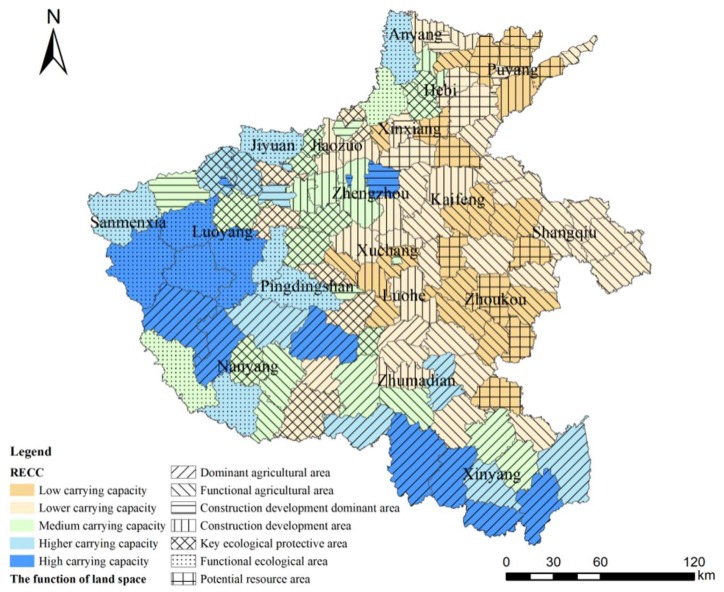
Evaluation results of RECC and land space development zoning in Henan province.

The agricultural production area is mainly composed of the Nanyang Basin and Huanghuaihai Plain (Figure 7). In this area, the terrain is flat, water and soil resources are abundant, and the cultivated land area is large. Moreover, the RCC is strong. This is an important grain production area of the province and a modern agricultural demonstration area. As an important grain producing area in China, Henan Province plays an important role in the development of the province and needs to properly protect and optimize agricultural functional areas. Therefore, in terms of zoning construction, the Huanghuaihai Plain and the Nanyang Basin should be the principal focus for protecting cultivated land quality and constructing high-standard basic farmland; then, we should focus on building the functional area for grain production. Secondly, according to local conditions, ecological compound agriculture should be planted, and then the characteristic agriculture advantage area with a reasonable layout should be formed. Finally, there should be a reasonable arrangement of the land for rural living and non-agricultural production. Meanwhile, we should strengthen the guidance of rural planning and promote village rectification and new rural constructions in an orderly manner.

The ecological protection area is mainly situated in the west of Henan, the western part of the northern Henan area, the middle and lower reaches of the Yellow River, and the south of the Qinling Mountains–Huaihe River (Figure 7). The topography and landforms in the ecological protection area are mainly mountainous and hilly, as well as being rich in natural resources and superior in terms of the ecological environment; thus, this is an important ecological conservation area in Henan Province. As a prohibited development zone and a restricted development zone, the ecological protection area should focus on improving environmental quality and protecting ecological barriers and then constructing the ecological security pattern of “three screens, four corridors, and one district”. In addition, we should implement the classification control of the ecological protection area and create an urban development layout that is ecological and livable.

The construction development area is the political and economic center of the province, which mainly includes Zhengzhou, Luoyang, Xinxiang, Jiaozuo, Xuchang, and other cities (Figure 7); this is also the area with the largest urban space and population density. However, the main construction development area has poor resource and environmental carrying capacity, its potential resources are scarce, and the environmental pollution problems are prominent. Therefore, the region should rationally develop economic industries under the premise of protecting the ecological environment. Meanwhile, according to the evaluation results of the carrying capacity and the development intensity requirements, the region should reasonably delimit its urban development boundary and then promote the intensive and efficient development of urban space.

## 5. Discussion

### 5.1. Distribution of Carrying Capacity and Functional Areas of Henan Province

In order to study the distribution characteristics of RECC and the function of land space in Henan Province, and to propose suitable spatial optimization schemes, we need to analyze the regional numbers with different RECC levels in Henan Province. Then, we need to diagnose the quantity status of each functional area under different RECC levels in Henan Province.

**Figure 8:**
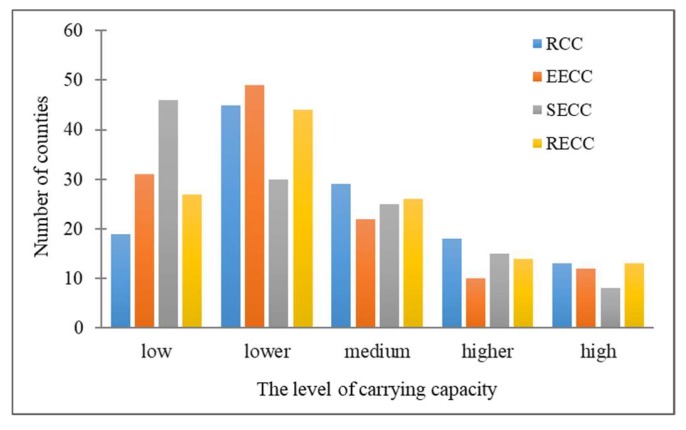
Number of counties at different levels of RCC, EECC, SECC, and RECC in Henan Province.

A previous study published in 2019 also focused on the RECC of Henan Province [55]. The study found that, from 2005 to 2015, the RECC of each city in Henan Province varied greatly, and the spatial differentiation characteristics were obvious. In addition, there were many areas with a medium RECC in 2015. In our study, as shown in Figure 8, from the perspective of level distribution, most of the carrying capacity levels are distributed at the lower and medium levels. On the contrary, the proportions of the higher and high levels of carrying capacity are lower than those at the other carrying capacity levels, which shows that the pressure on the resource and environmental systems within most areas of Henan Province is high. Therefore, there are similarities between the two studies. Additionally, the carrying pressure of Henan Province decreases from southeast to northwest. This also accords with the economic and population distribution pattern of Henan Province. In our study, we conjectured that a large population scale leads to greater economic development and more efficient human activities, which increases the unsustainable resource utilization and resource consumption and further causes the decline of the RECC.

**Figure 9:**
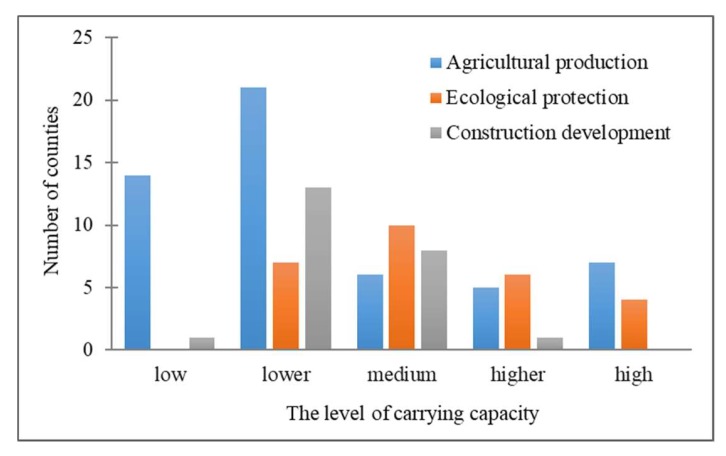
Number of functional areas of different levels of RECC in counties.

The current study of land space function in Henan Province is mainly aimed at the city level. There are few studies that focus on the county level [56]. Meanwhile, the existing studies of land space function have paid less attention to the relationship between land space function and RECC. As shown in Figure 9, this study finds a close relationship between the land space function and RECC at the county level of Henan Province. For instance, according to this study, the agricultural production areas were mainly distributed at the level of low RECC and lower RECC. This result demonstrates that we should protect and rationally use agricultural resources and prohibit abuse and unreasonable development. In contrast, the ecological protection areas were mainly distributed at the level of medium RECC and higher RECC, which shows that most of the ecological protection areas in Henan Province are at a better load level. However, there are still some areas at lower carrying levels that require greater attention and protection in the future. Additionally, construction development areas are mainly distributed at the level of lower RECC and medium RECC, and it is shown that the area should rationally develop economic industries under the premise of protecting resources and the ecological environment.

### 5.2. Relationship between Carrying Capacity and Land Space Development Zoning

In recent years, some researchers have mainly focused on single factor evaluations of carrying capacity or regional carrying capacity analyses [57,58,59] but have not considered the comprehensive capacity of resources and the environment and have not provided useful support for spatial planning work. Moreover, due to the obvious differentiation of resources in China’s national territory, the different regions used as evaluation units to explore the spatial differences of RECC and then guide the layout of land space and economic development are the focus of current spatial planning research [60]. Therefore, in this study, the geographical spatial differentiation of RECC was illustrated, through which we could determine where the RECC was higher and lower in Henan Province. It can be seen from the results that the spatial differences of RECC in the regional space are obvious. In addition, the resources in the high carrying capacity area are better, and the social–economic development causes less damage to the ecological environment. On the other hand, the low carrying capacity area has a large population density, and the resource and environmental development density are high, and therefore the carrying pressure is also relatively high. Thus, controlling the overly-rapid development of regional space based on the results of RECC and engaging in reasonable development under the premise of protecting the ecological environment are the key directions required to solve the problem of land space layout.

In addition, the development zoning of land space is an objective reflection of the law of regional spatial differentiation and is an important basis for formulating differentiated land space management policies. As an indispensable basis for optimizing the development layout of land space, RECC can provide an important role for the rational division of land space [21]. In our study, we related the RECC to the functional orientation of land space, which is different from the previous analysis of land space zoning [61]. Moreover, our study discussed the method of regional spatial division from the perspective of carrying capacity. Based on the explanation of the relationship between RECC and the functional type of land space, we used a three-dimensional magic cube evaluation model. In the three-dimensional cube’s model space, the RCC, EECC, and SECC, respectively, correspond to the agricultural production function, ecological protection function, and construction development function of the land space. Then, according to the results of RECC, the carrying level under different functions in the region can be judged in order to reasonably lay out the functional orientation of land space. The previous zoning results cannot fully reflect the resource and environmental carrying status in the region [43,62], so our study can compensate for the lack of carrying capacity in the traditional planning results and provide a reference for government departments to carry out research on the layout of national land space.

## 6. Conclusions

During the process of sustainable development, it is important to measure the relationship between the RECC and land space. Using a three-dimensional magic cube evaluation model, this article theoretically improved the shortcomings of traditional land space zoning methods and researched the layout of land space based on RECC levels. The results of this article prove that each county has different RECC and land space functional structures, thereby providing a decision-making basis for Henan Province to find differentiated paths of land space zoning. Based on our results, policy implications for the sustainable development and spatial planning of Henan Province were proposed. The main conclusions of this article are as follows:

(1) The spatial pattern of RECC showed an unbalanced carrying capacity situation in Henan Province. The county area with a high carrying capacity is mainly formed in a semi-circular distribution along the southwestern part of Henan Province. The resources and environment in this area are better, and the damage caused by social and economic development to the ecological environment is small. The county area with a low carrying capacity is mainly concentrated in the northeastern and central part of Henan Province. In this area, the population density is large, the density of resources and environmental development is high, and the carrying pressure is relatively high. Therefore, local governments need to put forward a scientific management program to solve the unbalanced problem of RECC. Specifically, the northeastern and central regions should take the protection of the ecological environment as their premise and change their economic development mode, while the southwestern region could use ecological advantages as its foundation and create a characteristic ecological industry system.

(2) The three-dimensional magic cube model can correlate RECC evaluation with land space zoning. In the three-dimensional space, the RCC, EECC, and SECC can correspond to the function of agricultural production, the function of ecological protection, and the function of construction development. Therefore, land space zoning based on the RECC results can prove whether the region’s layout is reasonable. The zoning results fully reflect the spatial patterns of RECC, which avoids subjectivity in planning.

(3) Using the three-dimensional magic cube model, the land space pattern of Henan Province can be divided into seven types of areas—agricultural dominant areas, agricultural functional areas, ecological key protective areas, ecological functional areas, construction development dominant areas, construction development areas, and potential resource areas—which can better reflect the spatial differentiation characteristics of the comprehensive index of RECC. Based on the differences of each functional area, local governments should implement different use control policies for different types of functional regions. In functional agricultural areas, the government should set up a “survival line” and then clarify the cultivated land protection area and scale of water resource development. In functional ecological areas, the government should set up “ecological lines”, clarify the scope of protected areas, and improve the level of ecological security. In construction development areas, the government should set up “security lines” that ensure the construction land necessary for economic and social development.

(4) Our study provides reference and application value for spatial planning. However, the selection of indicators depends to some extent on the knowledge and experience of experts in different disciplines and different fields. In addition, because the evaluation results may be affected by subjective factors, a reduction of the uncertainty caused by the results of the index analysis needs to be further developed in a future study.

## Figures and Tables

**Table 1:** Evaluation index system of the resource–environmental carrying capacity (RECC).

System	Criteria Layer	Indicators (Unit)	Serial Number	Weight	Attributes
RECC	Resource carrying capacity (RCC)	Area of construction land (hectares)	X_1_	0.0214	+
		Per capita constructive land area (m^2^/person)	X_2_	0.0407	+
		Per capita cultivated land (m^2^/person)	X_3_	0.0276	+
		Per capita forest land (m^2^/person)	X_4_	0.0484	+
		Total water resources (m^3^)	X_5_	0.1659	+
		Per capita water resources (m^3^/person)	X_6_	0.0591	+
		Per capita grain output (kg/person)	X_7_	0.0307	+
	Eco–environmental carrying capacity (EECC)	Percentage of ecological land coverage (%)	X_8_	0.0648	+
		Percentage of green land coverage (%)	X_9_	0.0785	+
		Atmospheric environment capacity (10,000 t/a)	X_10_	0.0316	+
		Water environment capacity (m^3^)	X_11_	0.0416	+
		COD emissions (t/a)	X_12_	0.0087	−
		Industrial SO_2_ emissions (t/a)	X_13_	0.0101	−
		dust emissions (t/a)	X_14_	0.0045	−
	Socio–economic carrying capacity(SECC)	Urbanization rate (%)	X_15_	0.0439	+
		GDP (10,000 yuan)	X_16_	0.0713	+
		Per capita GDP (yuan/person)	X_17_	0.0712	+
		Secondary and tertiary industries as percentage to GDP (%)	X_18_	0.0207	+
		GDP of per unit area(10,000 yuan/km^2^)	X_19_	0.1354	+
		Land consumption of 10,000 yuan GDP (hectare/10,000 yuan)	X_20_	0.0058	−
		Water consumption of 10,000 yuan GDP (m^3^/10,000 yuan)	X_21_	0.0039	−
		Population density (persons/km^2^)	X_22_	0.0142	−

Note: “+”means a positive index, “−”means a negative index.

**Table 2:** The division criteria of the RECC level.

Score Range of RECC	Classification Level
[Mean value + Standarddeviation, MAX]	4
[Mean value, Mean value + Standarddeviation]	3
[Mean value − Standard deviation, Mean value]	2
[MIN, Mean value − Standarddeviation]	1

**Table 3:** The land space development zoning principle.

The Function of Land Space	The Coordinates in the Cube	The Serial Number	The Type of Land Space Development	Note
Agricultural production	(4, b, c)	A1	Dominant agricultural area	b ≥ 1, c ≤ 3
	(3, b, c)	A2	Functional agricultural area	b ≥ 1, c ≤ 2
Ecological protection	(a, 4, c)	E1	Key ecological protective area	a ≥ 1, c ≤ 3
	(a, 3, c)	E2	Functional ecological area	a ≥ 1, c ≤ 2
Construction development	(a, b, 4)	C1	Construction development dominant area	a ≥ 1, b ≤ 3
	(a, b, 3)	C2	Construction development area	a ≥ 1, b ≤ 2
Potential resource	(a, b, c)	R1	Potential resource area	a, b, c∈ (1, 2)
